# Central Venous Blood Gas Analysis: An Alternative to Arterial Blood Gas Analysis for pH, PCO_2_, Bicarbonate, Sodium, Potassium and Chloride in the Intensive Care Unit Patients

**DOI:** 10.5005/jp-journals-10071-23176

**Published:** 2019-06

**Authors:** Mubina Begum Bijapur, Nazeer Ahmed Kudligi, Shaik Asma

**Affiliations:** 1-3 Department of Anaesthesiology and Critical Care, Al Ameen Medical College, Vijayapura, Karnataka, India

**Keywords:** Agreement, Arterial blood gas analysis, Central venous blood, Correlation, Electrolytes

## Abstract

**Aims:**

Arterial blood gas (ABG) analysis is a frequently ordered test in intensive care unit (ICU) and can analyze electrolyte in addition to pH and blood gases. Venous blood gas (VBG) analysis is a safer procedure and may be an alternative for ABG. Electrolyte estimation by auto analyzer usually takes 20–30 minutes. This study was aimed to investigate the correlation of pH, PCO_2_, bicarbonate, sodium, potassium, and chloride (electrolytes) between ABG and central VBG in ICU patients.

**Materials and methods:**

This was a prospective observational study conducted in medical college hospital ICU. Adult patients requiring ABG and electrolyte estimation as a part of their clinical care were consecutively included in the study. Patients having any intravenous infusion or who were pregnant were excluded. Venous samples were taken within 2 minutes of arterial sampling from *in situ* central line. Data were analyzed using Bland-Altman methods.

**Results:**

A total of 110 patients' paired blood samples were analyzed. The mean difference between arterial and central venous values of pH, PCO_2_, bicarbonate, sodium, potassium, and chloride was 0.04 units, –5.84 mm Hg, 0.89 mmol/L, –1.8 mEq/L, –0.04 mEq/L, and –0.89 mEq/L, respectively. The correlation coefficients for pH, PCO_2_, HCO_3_^−^, sodium, potassium, and chloride were 0.799, 0.831, 0.892, 0.652, 0.599 and 0.730, respectively. Limits of agreement (95%) were within acceptable limits.

**Conclusion:**

Central venous pH, PCO_2_, and bicarbonate may be an acceptable substitute for ABG in patients admitted in the ICU. However caution should be exercised while applying electrolyte measurements.

**How to cite this article:**

Bijapur MB, Kudligi NA, Asma S. Central Venous Blood Gas Analysis: An Alternative to Arterial Blood Gas Analysis for pH, PCO_2_, Bicarbonate, Sodium, Potassium and Chloride in the Intensive Care Unit Patients. Indian J Crit Care Med 2019;23(6):258–262.

## INTRODUCTION

Arterial blood gas (ABG) analysis is a commonly performed test to evaluate respiratory and acid base status in critically ill patients admitted to intensive care unit (ICU). Though ABG analysis is rapid and reliable, the arterial puncture carries a risk of hemorrhage and other vascular complications,^1^ which is painful and no longer necessary for diagnosing respiratory failure because of widespread use of pulse oximetry for measuring oxygen saturations. For these and other reasons such as ease of collection, the peripheral venous blood gas (VBG) analysis is increasingly being used as a replacement to the ABG especially in the emergency department. Sodium, potassium, and chloride (electrolytes) abnormalities^2^ are also one of the common causes of morbidity and mortality in ICU patients and are conventionally measured by auto analyzers available in hospital's central laboratories. Typically, an average turnaround time of 20–30 minutes is noted in acute care laboratories of most tertiary care hospitals. Point of care testing for electrolytes is available, but cost is a major deterrent for their utilization in developing countries.

Earlier studies have shown good correlation between ABG and VBG values with respect to pH, PCO_2_, and bicarbonate in adult patients presented to the emergency department and ICU.^3,4^ Studies involving ICU patients have shown good correlation for potassium measured between ABG machine and point of care analyzers.^5,6^ However, there is limited information regarding relationship between the venous and arterial values for pH, PCO_2_, bicarbonate, and electrolytes in critically ill patients. Central venous catheter (CVC) is inserted for central venous pressure measurement, sampling of blood for investigation, VBG analysis, and drug administration. The aim of the study was to determine the extent of agreement between arterial and central venous samples for pH, PCO_2_, and bicarbonate along with electrolytes in ICU patients.

## SUBJECTS AND METHODS

After receiving approval from hospital's Research Ethical Committee, this prospective observational study was done on 110 adult patients of both sexes admitted to ICU at a medical college hospital. After having the study explained, a verbal consent was obtained from either the patient or the relative. The patients requiring blood gas and electrolyte analyses as a part of their clinical care were included consecutively in the study. Pregnant patients and those on intravenous infusion were excluded from the study. When an ABG analysis and electrolytes deemed to be necessary as part of patient management, a central venous sample was drawn in minimally heparinized plastic syringe within 2 minutes of sampling of arterial blood (0.5–1 mL) from radial or dorsalis pedis artery. The samples were taken before the initiation of any form of treatment. Either a nurse or resident doctor withdrew and prepared samples for analysis as per ICU protocol. All of the samples were analyzed as quickly as possible using the ICU based radiometer, ABL80 FLEX analyzer, which was calibrated according to standard quality assurance protocols.

Statistical analysis was carried out by using Med Calc 9.0.1, Systat 12.0, and R environment version 2.11.1 software. The Bland-Altman method^7^ was used to assess agreement between arterial and venous measurements of pH, PCO_2_, bicarbonate and electrolytes. The arterial minus venous (A-V) difference versus average value (A+V)/2 was plotted. Means, standard deviations (SD), and 95% prediction intervals (limits of agreement) of the A-V differences were reported. In addition, Pearson's product moment correlation coefficients between arterial and central venous values were reported. Correlation coefficient values range from being negatively correlated (-1) to uncorrelated (0) to positively correlated (+1) (0.0 is no correlation, + 0.3 is weakly positive, +0.5 is moderately positive, +0.8 is strongly positive, and +1.0 is perfectly positive). Linear regression was used to establish equations for estimation of arterial values from central venous values.

## RESULTS

The study involved a total of 110 patients [70 males (63.6%) and 40 females (36.4%), mean ± SD and age of 40.91 ± 16.21 years]. A total of 110 paired blood samples were included in the study. The presenting diagnoses of the patients were: poisoning (27.27%), neurological (20.9%), postsurgical (13.63%), trauma (10.90%), respiratory (9.09%), cardiovascular (5.45%), and sepsis and other infectious diseases (6.36% each). Arterial values were as follows: pH (6.99–7.58), PCO_2_ (17–134 mm Hg), HCO_3_^-^ (9.2–43.5 mEq/L), Na^+^ (113–173 mEq/L), K^+^ (1.7–7.3 mEq/L), and Cl^-^ (79–139 mEq/L). Corresponding venous values were: pH (6.96–7.55), PCO_2_ (18–146 mm Hg), HCO_3_^-^ (9.6–43.2 mEq/L), Na^+^ (116–172 mEq/L), K^+^ (2.0–7.6 mEq/L), and Cl^-^ (82–134 mEq/L).

Table 1 shows the mean values (SD), arterial minus central venous differences, Pearson correlation coefficients as well as Bland-Altman 95% limit of agreements (LOA) for pH, PCO_2_, bicarbonate and electrolytes between ABG and cVBG measurements. Arterial pH, PCO_2_, bicarbonate and electrolytes were significantly correlated with their central venous equivalents (*p* = 0.001 for all; correlation coefficients = 0.799, 0.831, 0.892, 0.652, 0.599, 0.730, respectively).

**Figs 1A to C:**
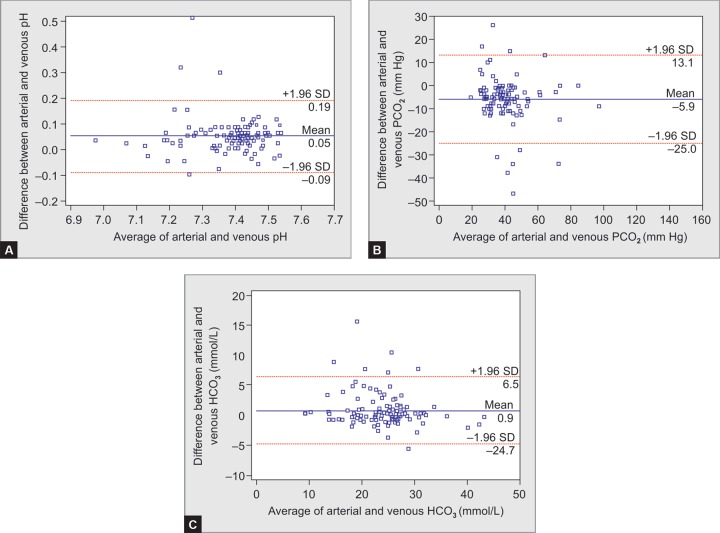
Bland-Altman plot of arterial and venous (average vs difference) (A) pH, (B) PCO_2_, and (C) HCO_3_^−^

**Figs 2A to C:**
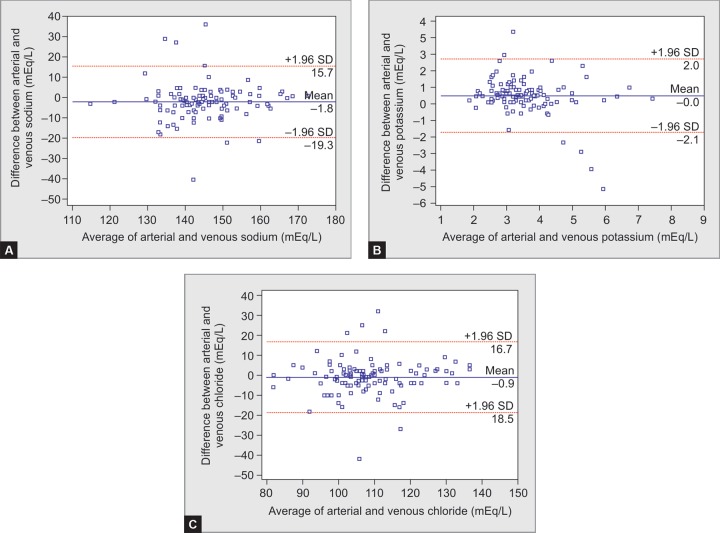
Bland-Altman plot of arterial and venous (average vs difference) (A) sodium; (B) potassium; and (C) chloride

A Bland-Altman plot of arterial and central venous blood for pH, PCO_2_, bicarbonate, sodium, potassium, and chloride with the 95% LOA (dotted lines) of A-cV difference is shown in Figure 1 and Figure 2 (pH: - 0.09–0.19; PCO_2_: -25–13.1; HCO_3_^-^ : -4.7–6.5; Na^+^: -19.3–15.7; K^+^: -2.1–2.0, and Cl^-^ : -18.5–16.7). Regression equations were derived to predict ABG values from cVBG measurements for pH, PCO_2_, HCO_3_^-^, sodium, potassium and chloride and their coefficient of determination (R^2^) as follows:

Arterial pH = 1.277 + 0.821 × venous pH (R^2^ = 0.638)

Arterial PCO_2_ = 9.293 + 0.913 × venous PCO_2_ (R^2^ = 0.690,)

Arterial HCO_3_^-^ = 9.293 + 0.913 × venous HCO_3_^-^ (R^2^ = 0.795)

Arterial Na^+^ = 54.667 + 0.633 × venous Na^+^ (R^2^ = 0.425)

Arterial K^+^ = -0.114 + 0.968 × venous K^+^ (R^2^ = 0.358)

Arterial Cl^-^ = 0.896 + 0.757 × venous Cl^-^ (R^2^ = 0.532)

## DISCUSSION

Arterial blood gas is an important part of assessment of clinical status and progress of critically ill patients with presumed blood gas and acid base imbalance. However, it may not always be possible to obtain arterial samples. Arterial punctures and long term *in situ* arterial catheters pose a small but significant risk of complications.^8^ Besides this, electrolyte abnormalities are also one of the common causes of morbidity and mortality in critically ill patients^2^ and are conventionally measured from venous blood serum by central laboratory analyzer which takes about 20–30 minutes. Patients in ICU often have *in situ* central venous catheters, and central VBG analysis may be a safer alternative to ABG analysis for determining acid base status and electrolytes level, thereby reducing the need for frequent invasive arterial sampling and turnaround time of electrolytes estimation.

The study showed strong correlation between arterial and central venous values for pH, PCO_2_, and bicarbonate, while it was moderate for sodium, potassium, and chloride. Arterial and venous values for pH correlated satisfactorily (r = 0.799). The mean arterial minus central venous difference was 0.04 (SD 0.07) with 95% LOA ranging from - 0.09 to 0.19, which was moderate enough to allow for substitution (Fig. 1A). This is consistent with the findings of studies by Tregger et al.,^3^ Awasthi et al.,^4^ Middleton et al.,^9^ and Bo Ra kim et al.,^10^ which have shown a mean A-V difference for pH ranging from –0.04 to 0.053.

With respect to PCO_2_ values, ABG and cVBG values correlated satisfactorily (r = 0.831), but their 95% LOAs were too wide to allow substitution (-25–13.1). Given that the blood gas values should be interpreted in the context of the individual patient's clinical status and that frequently arterial blood gases are obtained to help assess a patient's course, central venous PCO_2_ largely should be able to replace arterial PCO_2_ in the most clinical circumstances.

**Table 1:** Summary of arterial and central venous blood gas values (n = 220)

*Parameter*	*ABG (Mean ± SD)*	*VBG (Mean ± SD)*	*A-V difference (Mean ± SD)*	*Correlation coefficient (r value)*	*Bland-Altman 95% LOA*
pH	7.4±0.10	7.36±0.11	0.04±0.07	0.799	−0.09 to 0.19
PCO_2_ (mm Hg)	38.73±15.71	44.58±17.35	−5.84±9.77	0.831	−25 to 13.1
HCO_3_^−^ (mEq/L)	24.50±5.79	23.60±6.28	0.89±2.85	0.892	−4.7 to 6.5
Na^+^ (mEq/L)	144.09±10.85	145.89±10.55	−1.8±8.94	0.652	−19.3 to 15.7
K^+^ (mEq/L)	3.51±0.99	3.55±1.25	−0.04±1.03	0.599	−2.1 to 2.0
Cl^−^ (mEq/L)	108.1±12.4	108.99±12.02	−0.89±8.98	0.730	−18.5 to 16.7

*ABG*, arterial blood gas; *VBG*, venous blood gas; *A-V*, arterial minus venous difference; *SD*, standard deviation; *LOA*, limits of agreement

Arterial blood gas and cVBG values for HCO_3_^-^ showed good correlation (r = 0.892). The mean arterial minus central venous difference for HCO_3_^-^ was 0.89 mmol/L, which was within clinically acceptable limits according to Tregger et al.^3^ and Rang et al.^11^ In this study, LOA (-4.7–6.5) was wide which was close to LOA by Tregger et al.^3^ (-4–2.4) who have concluded this limit as an excellent agreement between arterial and venous HCO_3_^-^. The venous pH is generally lower than arterial pH and venous PCO_2_ was generally higher than arterial PCO_2_, mean venous HCO_3_^-^ was unexpectedly higher than the mean arterial value. We conclude that value of HCO_3_^-^ was influenced more by CO_2_ level on which the calculation is based rather by the pH.

Arterial and central venous values of electrolytes moderately correlated (Table 1). Mean arterial minus venous difference were sodium: -1.8 (SD 8.94), potassium: -0.04 (SD 1.03), and chloride: -0.89 (SD 8.98), which is consistent with study by Awasthi et al.,^4^ which showed good correlation between both samples and low arterial minus venous differences for pH, bicarbonate, and electrolytes in group of ICU and critically ill patients. Johnston^5^ studied agreement between arterial and venous blood potassium in patients with cardiac arrest. It was found that mean difference between each pair of arterial and venous potassium was 0.106 mmol/L. Our study revealed that a mean difference of 0.04 mmol/L is comparable to the author's analysis. Nanda et al.^12^ found the mean values of arterial sodium and potassium were lower than venous sodium and potassium, which is consistent with the findings of present study. Wongyingsinn et al. observed a good correlation between arterial and venous potassium and stated that arterial potassium can replace measurement of venous potassium.^13^ Flegar Mestric et al. observed that electrolytes measured in whole blood by point of care analyzer were comparable to electrolytes measured in plasma or venous serum samples.^14^ Jain et al. observed that there was no significant difference between potassium measured in ABG analyzer and potassium measured by routine chemistry auto analyzer.^15^

In the present study, which showed excellent correlation for acid base status with moderate correlation for electrolytes, this could become promising to use VBG analysis electrolytes along with pH, PCO_2_, and bicarbonate in the place of ABG and serum electrolytes in early stages of resuscitation in emergency department and ICU patients. This can reduce serious complications associated with long term arterial catheterization and time required for electrolyte analysis. Due to good correlation and acceptable mean differences, the present study can suggest that changes in venous values would reflect changes in the corresponding arterial values and therefore can be used for trending purposes.

Limitations of the study included patients with diverse pathological conditions with abnormal blood gas and electrolyte values. Further study of the differences in the clinical decision making based on VBG analysis for acid base status and electrolyte levels from a specific patient population with a likelihood of abnormal blood gases and electrolytes is necessary before recommending routine use of an abnormal VBG.

## CONCLUSION

The study showed good correlation for pH, PCO_2_, bicarbonate values with moderate correlation for electrolytes. These results suggest that venous values may be an acceptable substitute for arterial measurement in many clinical settings encountered in ICU obviating the need for repeated arterial sampling. However, caution should be exercised while applying electrolyte measurements though it reduces the electrolyte estimation time and is cost effective. Further research is required to establish their accuracy with respect to electrolyte values.
